# Communities, birth attendants and health facilities: a continuum of emergency maternal and newborn care (the global network's EmONC trial)

**DOI:** 10.1186/1471-2393-10-82

**Published:** 2010-12-14

**Authors:** Omrana Pasha, Robert L Goldenberg, Elizabeth M McClure, Sarah Saleem, Shivaprasad S Goudar, Fernando Althabe, Archana Patel, Fabian Esamai, Ana Garces, Elwyn Chomba, Manolo Mazariegos, Bhala Kodkany, Jose M Belizan, Richard J Derman, Patricia L Hibberd, Waldemar A Carlo, Edward A Liechty, K Michael Hambidge, Pierre Buekens, Dennis Wallace, Lisa Howard-Grabman, Suzanne Stalls, Marion Koso-Thomas, Alan H Jobe, Linda L Wright

**Affiliations:** 1 Department of Community Health Sciences, Aga Khan University, Karachi, Pakistan; 2 Department of Obstetrics and Gynecology, Drexel University, Philadelphia, Pennsylvania, USA; 3 Department of Statistics and Epidemiology, Research Triangle Institute, Durham, North Carolina, USA; 4 KLE Research Foundation, Jawaharlal Nehru Medical College, Belgaum, India; 5 Institute for Clinical Effectiveness and Health Policy, University of Buenos Aires, Buenos Aires, Argentina; 6 Department of Pediatrics, Indira Gandhi Government Medical College, Nagpur, India; 7 Department of Pediatrics, Moi University, Eldoret, Kenya; 8 San Carlos University, Guatemala City, Guatemala; 9 Department of Pediatrics, University of Zambia, Lusaka, Zambia; 10 ECLAMP, Guatemala City, Guatemala; 11 Department of Obstetrics and Gynecology, Christiana Health Care, Delaware, USA; 12 Partners Health Organization, Boston, Massachusetts, USA; 13 Department of Pediatrics, University of Alabama at Birmingham, Birmingham, USA; 14 Department of Pediatrics, Indiana University, Indianapolis, USA; 15 Pediatric Nutrition, University of Colorado, Denver, USA; 16 Tulane School of Public Health and Tropical Medicine, New Orleans, USA; 17 TRG Incorporated, Alexandria, USA; 18 Albuquerque, USA; 19 Eunice Kennedy Shriver National Institute of Child Health and Human Development, Bethesda, USA; 20 Department of Pediatrics, Cincinnati Children's Hospital, Cincinnati, USA

## Abstract

**Background:**

Maternal and newborn mortality rates remain unacceptably high, especially where the majority of births occur in home settings or in facilities with inadequate resources. The introduction of emergency obstetric and newborn care services has been proposed by several organizations in order to improve pregnancy outcomes. However, the effectiveness of emergency obstetric and neonatal care services has never been proven. Also unproven is the effectiveness of community mobilization and community birth attendant training to improve pregnancy outcomes.

****Methods/Design**:**

We have developed a cluster-randomized controlled trial to evaluate the impact of a comprehensive intervention of community mobilization, birth attendant training and improvement of quality of care in health facilities on perinatal mortality in low and middle-income countries where the majority of births take place in homes or first level care facilities. This trial will take place in 106 clusters (300-500 deliveries per year each) across 7 sites of the Global Network for Women's and Children's Health Research in Argentina, Guatemala, India, Kenya, Pakistan and Zambia. The trial intervention has three key elements, community mobilization, home-based life saving skills for communities and birth attendants, and training of providers at obstetric facilities to improve quality of care. The primary outcome of the trial is perinatal mortality. Secondary outcomes include rates of stillbirth, 7-day neonatal mortality, maternal death or severe morbidity (including obstetric fistula, eclampsia and obstetrical sepsis) and 28-day neonatal mortality.

**Discussion:**

In this trial, we are evaluating a combination of interventions including community mobilization and facility training in an attempt to improve pregnancy outcomes. If successful, the results of this trial will provide important information for policy makers and clinicians as they attempt to improve delivery services for pregnant women and newborns in low-income countries.

**Trial Registration:**

ClinicalTrials.gov NCT01073488

## Background

### Research justification and relevant literature

More than half a million maternal deaths, over 3 million stillbirths and 3 million early neonatal deaths occur each year worldwide, the majority in South Asia and sub-Saharan Africa[1-4]. Delivery complications (prolonged labor, preeclampsia, maternal infection and obstetric hemorrhage) are responsible for half of all maternal deaths, one-third of stillbirths and one-quarter of neonatal deaths [5-9]. Intrapartum complications are also responsible for maternal morbidity, e.g., hemorrhage and obstetric fistulae, as well as childhood disability and long-term impairment [10-12]. Pregnancy complications, which are not easily predicted, usually first become apparent during labor and often require timely facility-based management to avert death and severe morbidity [13].

Emergency Obstetric and Neonatal Care (EmONC) requires a skilled birth attendant with the ability to provide parenteral medications (e.g., antibiotics, oxytocics, anticonvulsants); carry out procedures (e.g., manual removal of the placenta, forceps/vacuum deliveries); perform blood transfusions, cesarean sections and provide newborn care/resuscitation [14]. Despite established interventions, the majority of maternal and neonatal deaths occur due to a lack of access to life-saving services [15,16]. Women most likely to experience obstetrical emergencies are those with least access to appropriate services [17,18]. The medical model of maternal/newborn survival, with a linear path between recognition of intrapartum complications, stabilization/referral and receipt of good quality care, is complicated by contextual and cultural factors which may pose intractable barriers to access [17].

Current efforts to reduce peripartum deaths focus on skilled delivery attendance for every birth backed up by emergency obstetric care and facility-based delivery [19,20]. However, 60 million births currently occur outside facilities, 52 million without skilled attendance [21]. It is unlikely that countries with the majority of these deliveries will be able to establish systems to implement wide-scale skilled birth attendance in the near future. Equally doubtful is their ability to accommodate a large-scale shift in delivery site to facilities providing comprehensive delivery care in the near future [22]. Reducing peripartum morbidity and mortality will require solutions that take into account the scarce resources and inadequate infrastructure in countries with the worst indicators.

Substantive evidence for the reduction of intrapartum deaths focuses on individual interventions: pharmacologic management, community-participatory approaches, and health system interventions [15,16,23]. The problem does not appear to be lack of innovation but of appropriate, sustainable provision of these services to those who need them most [24]. Programs have used various combinations of these interventions successfully [13,22,25,26]; however, these combined programs have not been scientifically evaluated to determine whether they are effective in reducing intrapartum deaths.

### A continuum of services to reduce delays in emergency obstetric and newborn care

A successful model for reduction of intrapartum mortality must span care across time and place, including families, communities and providers of delivery and related services, with an emphasis on coverage and quality of care as well as functional linkages between the various levels of care [27].

#### Stakeholder Participation

The Alma Ata declaration reaffirmed the goal of "Health for All" and identified primary health care as the key to attaining this goal [28]. The declaration was based on the premise that, within communities, individuals and groups had to be part of the planning, implementation and appraisal efforts to meet their own needs on a sustained basis [29]. The emphasis on multi-sector and community involvement was lost as a selective model of primary health care was implemented [30]. Thirty years later, despite gains in many health outcomes, deaths around the time of delivery have proven to be difficult to reduce. There is increasing recognition that broad ownership of health and health systems may be one of the keys to improving peripartum outcomes.

Existing health systems, as service providers and regulators, are major stakeholders for improving maternal newborn health (MNH). Programs to bring about sustained change in MNH outcomes should reinforce national commitment [31]. Countries that have prioritized MNH at a national level, e.g. Rwanda and Nepal, have made major strides despite substantial challenges [32-34].

Successful community participatory approaches have shown reductions in maternal/neonatal mortality in Bolivia, India, Bangladesh and Nepal and provide evidence for engaging another key stakeholder, the community [35-39]. A range of approaches has been described as community mobilization/participation and can be differentiated on the actual level of community involvement. Community ownership and capacity to act independently increase the likelihood of sustained change [40]. A promising approach is one that builds on inherent community resources; this model has the advantage of being self-sustaining [41,42]; improving outcomes by capitalizing on existing community strengths and focusing on resources that can be leveraged to improve health [43]. Existing skills and resources which lead to better outcomes can be ascertained by identifying individuals who have solved problems despite being at greatest risk of a bad outcome [44].

#### Deliveries at home and primary facilities

Changing the behavior of families and communities during pregnancy, delivery and post-partum and reducing demand-side barriers to health service by addressing context-specific delays can improve outcomes in populations where the majority of deliveries take place at home or in primary facilities [15,45,46].

Interventions that have been proven effective or have strong conceptual underpinnings include improvements in domiciliary delivery practices such as hand-washing, use of a clean home delivery kits and/or clean blade to cut the cord [15]; birth preparedness, e.g. availability of funds, transport mechanisms and comprehensive obstetric care facilities[47]; and early recognition of and appropriate responses to complications[22,48]. Traditional birth attendants (TBA's) remain the main provider of delivery care especially in settings where mortality rates are high [21]. Despite the lack of evidence supporting TBA training as a single intervention, there are data to support the inclusion of trained TBA's within an improved health care system [16,49-51].

#### Quality of emergency obstetric and newborn care

Ideally, best practices based on current international standards should be available to all women presenting with obstetrical emergencies [12,52]. EmONC packages which include intrapartum monitoring may improve newborn survival (neonatal mortality reduction 25-75%) and reduce maternal mortality [24,52,53]. The challenge in settings with the worst intrapartum outcomes is to appropriately implement EmONC packages. Efforts to improve quality of care have focused on training including in-service training and obstetric simulations and drills [54,55]. Another effective tool for improving quality of care is the use of perinatal audits to institute changes or solutions for problems that caused fatality, with each cycle building on the previous one [56].

### Study rationale and objectives

An underlying premise of this study is that EmONC teams consisting of local residents and health care providers can develop and implement comprehensive interventions to address limited access to quality emergency obstetric and neonatal care. The EmONC trial will test the hypothesis that implementing these locally-driven processes can lead to a substantial reduction in poor perinatal outcomes in intervention compared to control locations with standard care. These EmONC teams will work within their community and with the existing health care system to reduce adverse pregnancy outcomes through a broad intervention which includes community mobilization to strengthen community capacity to identify and address barriers to improved MNH and drive client-oriented emergency obstetrical and neonatal care; community-based training to recognize prolonged labor, infection, preeclampsia and hemorrhage, and the use of appropriate stabilization methods that can be employed in homes and in first level care facilities; and improvement of quality of care in existing health facilities.

## Methods/Design

### Study Design

This community-based, two-arm cluster-randomized controlled trial will evaluate the impact of an EmONC package introduced by EmONC cluster teams in intervention clusters compared to standard care in control clusters. The study includes 106 clusters (defined as distinct geographic areas with approximately 300-500 births per year) and encompasses all deliveries therein. All clusters have pre-existing independent maternal-newborn health registry systems, i.e. independent, prospective, population-based active surveillance mechanisms to assess rates of stillbirth, neonatal and maternal mortality, on which the outcome assessment will be based.

### Study Outcomes

The primary outcome of the study is perinatal mortality, defined as the composite rate of stillbirth and 7-day neonatal mortality per 1000 births. We will collect outcome data on all births at 20 weeks gestation and greater, but the primary outcome will include events for those pregnancies that occur at ≥28 weeks or ≥1000 g. Secondary outcomes will include stillbirths, 7-day neonatal mortality, maternal death or severe morbidity (including obstetric fistula, eclampsia and obstetrical sepsis) and 28-day neonatal mortality. Other outcomes of interest include rates of transport to hospital of both mothers and newborns.

### Enrollment - Trial sites

The study clusters are located at 7 sites in six countries. (Table 1) All of these sites are research units of the Global Network (GN). From 2005 to 2007, five of the seven sites participated in a cluster-randomized trial of WHO Essential Newborn Care and Neonatal Resuscitation, the GN's FIRST BREATH trial [57].

**Table 1:** EmONC trial study sites

Country	Argentina	Guatemala	India		Pakistan	Kenya	Zambia
Site	Corrientes	Chimaltenango	Belgaum	Nagpur	Thatta	Western Province	Kafue
Clusters (n)	6	10	20	20	24	16	10
Total deliveries (n)^a^	2.541	2,518	19,529	4,344	10,404	8,436	6,374
Home deliveries^a ^(%)	1	72	19	14	59	67	57

^a ^The number of total deliveries and percentage of home deliveries are based on data collection over a 1-year period prior to the implementation of the EmONC trial for all sites except Chimaltenango, Guatemala and Nagpur, India, where delays in the approval process limited prior data collection to six months.

### Interventions in intervention sites

The intervention is based on best practices from existing research and programmatic experience by the GN's EmONC Trial investigators, comprised of obstetricians, neonatologists and public health professionals well versed in and/or practicing MNH in low-resource settings. All activities detailed below are implemented throughout the intervention areas.

#### EmONC teams' goals

At each site, a Country Advisory Group and Cluster Teams in each intervention cluster seek to accomplish the following:

1. Work in partnership with the local health authorities.

2. Implement an organized process of community mobilization, and social and behavioral change based on a "Community Action Cycle" that strengthens communities' capacity to organize, explore MNH issues and set priorities, plan, implement, monitor, evaluate and share effective strategies and systems that increasingly improve EmONC care and MNH outcomes.

3. Analyze preventable causes of peripartum mortality and existing community resources for EmONC, including quality evaluation of the MNH care services.

4. Assist communities to identify and leverage available resources to address barriers to EmONC (e.g., communications and transport for EmONC).

5. Identify and assess capacity of available EmONC referral hospitals, work with hospital staff to accept transfers, perform appropriate cesarean sections/other procedures and review/maintain quality of care. Actively engage community members as advocates for improvement in quality of care.

6. Identify/train all community birth attendants/health workers, as appropriate, to (a) identify need for referral for severe maternal/newborn illnesses (b) facilitate transfer to an EmONC facility by identifying and arranging for locally available and sustainable methods of transportation (c) stabilize the woman/newborn prior to/during transport () communicate with hospital staff for timely and effective transport.

7. Establish a quality assurance review in each cluster to review each maternal, fetal or neonatal death to determine if it was preventable and how to prevent similar deaths in the future.

#### EmONC team tools

To accomplish the components of the intervention described above, master trainers will facilitate central and regional training of 1 to 2 Country trainers in each of the following areas: (1) community mobilization; (2) birth attendant training, and (3) EmONC referral facility improvement.

##### Community Mobilization

Sustainable community mobilization must move beyond raising community awareness about an issue or persuade people to engage in predetermined activities. The Community Action Cycle (CAC), a comprehensive strategy, empowers communities to take charge of problems within their own context. The CAC has seven key phases: 1) Prepare to mobilize - the methodology to work with communities is designed, the study teams are established and trained, and they identify and train community facilitators; 2) Organize the community for action- the team enters the community, establishes credibility; raises community awareness about MNH; and works with communities to identify and invite those most likely to be affected by and interested in MNH issues to organize "core groups"; 3) Explore MNH issues and set priorities -core group members explore MNH problems and existing practices in their core groups and with the broader community and set priorities based on what they learn; 4) Plan together- core group members engage in a planning process with community leaders and resource people including health providers to develop a Community Action Plan and establish coordinating and monitoring mechanisms; 5) Act together - the community implements their plan and monitors progress, adjusting course as necessary; 6) Evaluate together- the community evaluates results, shares what it has learned, and prepares to begin the cycle again; 7) Prepare to scale-up - the community and/or the program team expands the approach to other communities.

Individuals from outside the community must establish relationships with communities built on mutual respect and allow the community to solve their problems in the most contextually appropriate way. The CAC works well with HBLSS as well as with efforts to strengthen of facility-based services.

##### Birth Attendant Training

Home Based Life Saving Skills (HBLSS), developed by the American College of Nurse Midwives, is a family/community-based program to reduce maternal/neonatal mortality with emphasis on basic life saving care at the community level; reduction in delays in reaching facilities and supporting birth preparedness involving key decision makers. HBLSS is a competency-based training intervention for community women and men, focusing on practices that are safe, feasible and acceptable to the community. HBLSS emphasizes community involvement during the administration of a 12-item curriculum of preventive/life saving skills. The pictorial HBLSS 'Take Action' cards are particularly useful in communities with low literacy levels. The core curriculum is complemented by activities to develop emergency transportation systems and involve community leaders, particularly men [58]. We emphasize that the entire community, including traditional birth attendants, received HBLSS training.

##### Improvement in Quality of Care at Facilities

The facility training utilized curriculum adapted from Jhpiego which includes a training curriculum and practice sessions in the following areas: emergency preparedness, essential newborn care, obstetrical care. The curriculum includes training on drills aimed at improving facility responses to emergency conditions such as hemorrhage and eclampsia, which are then to be conducted in-country, as are regularly occurring audits for all maternal, fetal and neonatal deaths. The site facility trainer is expected to visit each EMONC facility to conduct assessments of the facility each 6 months, and hold training sessions aimed at improving maternity and newborn care on a regular basis.

##### EmONC team intervention and implementation

All the teams underwent a central, intensive two-week training of trainers (TOT) led by Master Trainers. Separate trainers were identified for each site to work in three major areas: EmONC Facility Improvement, HBLSS training and Community Mobilization. The TOT was followed by one week in-country training led by Country Trainers for key stakeholders and supported by Master Trainers at each site. Each team was provided all relevant materials for the CAC, HBLSS curriculum and the facility improvement methodology, as well as a common manual of operations for field implementation. Each team utilized the tools in the most contextually appropriate method for their site in order to achieve the study objectives.

### Interventions in control clusters

The Maternal Newborn Health (MNH) registry detailed below is the only study-related activity in the control clusters.

### Administrative Structures - The Global Network's Subcommittee for the EmONC Trial

**Figure 1:**
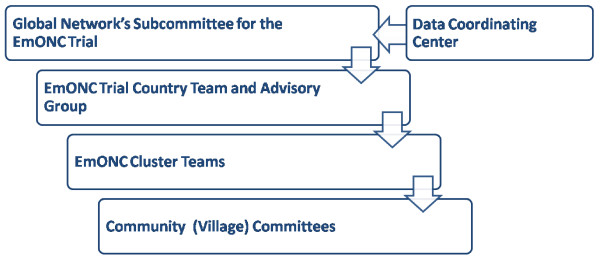
**Global Network EmONC Trial Organization**.

The Global Network for Women's and Children's Health Research (Global Network) is a multi-site trial funded by the National Institute of Child Health and Human Development (NICHD). The trial is supervised by the Global Network's EmONC Trial Subcommittee which consists of all site investigators, and the principal investigator and a senior statistician from the Data Coordinating Center (DCC) and the NICHD Director of the Global Network (Figure 1). The EmONC Trial Subcommittee convenes monthly by conference call and meets biannually to oversee study implementation, data analyses and publications.

### Administrative Structures - The EmONC Trial Country Team

At each site, the senior in-country investigators work with key members of the team responsible for implementation of the intervention and for the process data collection, including:

• **EmONC Country Coordinator: **responsible for overseeing the conduct of the study

• **Community Mobilization Trainer**: responsible for the training of Community Mobilization facilitators

• **Birth Attendant Trainer: **responsible for birth attendant training

• **EmONC Facility/Skilled Birth attendant Trainer**: physician responsible for leading the quality improvement and training at the EmONC facilities and leading the training for the skilled birth attendants at the level one health clinics.

### Administrative Structures - The EmONC Country Advisory Group

Each Country Team is supported by an EmONC Country Advisory Group consisting of the senior in-country investigator, EmONC Country Coordinator, a senior physician from a referral facility, three country trainers, a government health official, a community representative, a maternal representative and a birth attendant representative. The Advisory Groups facilitate buy-in for the intervention, assist in organizing trainings, death audits and community-based activities.

### Administrative Structures - EmONC Cluster Teams

The Cluster Teams are led by Cluster Coordinators and are responsible to oversee and coordinate all community mobilization activities and HBLSS training in their respective clusters. The EmONC Cluster Teams at each site meet once a month with the Country Team to discuss progress and obstacles in the implementation of the trial.

### Administrative Structures - Community (Village) Committees

These committees are formed through the CAC and are comprised of active community members who are responsible for coordinating implementation of the community's plan (e.g., improving transport, maintaining emergency funds, carrying out death audits, etc.) and monitoring results.

### Administrative Structures - The Data Coordinating Center

RTI International is the DCC for this trial. They are responsible for maintaining the central study database. In addition, they also generate descriptive statistics to monitor enrollment and retention, as well as process indicators for implementation of the study intervention.

### Informed Consent and Research Ethics

The study protocol and informed consent documents were reviewed and approved by the institutional review boards at all participating sites in as well as at the partner U.S. sites and DCC.

#### Informed consent was sought at two levels

**• ***Community and Health Facility Consent*: Agreement for study participation was sought from all participating communities and facilities in advance of randomization. Responsible community health authorities agreed to participate before beginning any activities. Communities were informed of their assignment to the control or intervention arm following randomization.

**• ***Pregnant women and their offspring: *Informed consent for data collection is sought from all pregnant women that are residents of or deliver within the study clusters.

#### Publications

The primary publication for the trial will follow CONSORT guidelines for randomized controlled trials. Criteria for authorship of all papers, presentations, and reports resulting from the study will conform to ICMJE standards.

### Quality Assurance

Multiple mechanisms are in place to assure the fidelity of the intervention and the quality of data collection. Cluster Coordinators, selected by the EmONC Country Team based on their skills, received training and certification on data collection, with refresher training conducted as needed. Cluster Coordinators directly monitor all aspects of the study intervention. The Country Team members visit the participating communities routinely. The DCC performs inter and intra-form edits to assure data consistency and monitors double-data entry. In addition, the DCC statisticians perform data quality reviews on an ongoing basis.

### Data Monitoring Committee, Interim Analyses and Stopping Rule

The NICHD-designated Data Monitoring Committee (DMC) reviews the data including enrollment, compliance, protocol violations, outcomes, and adverse events, at least every six months. Adverse events (including death, life-threatening events, and hospitalization) are monitored by onsite staff, submitted to the DCC and reported to the DMC.

### Statistical Design and Sample Size

The cluster-randomized trial was designed to test the impact of the intervention on perinatal mortality with the cluster as the unit of randomization and analysis. We will test the primary hypothesis that the EmONC will reduce the risk of perinatal mortality (measured as the total number of stillbirths and 7-day neonatal deaths per 1000 deliveries) with a permutation test conducted at the cluster level. Secondary analyses of this same hypothesis controlling for individual-level covariates will be conducted using extensions of the generalized linear model that account for the correlation within cluster. An assessment of whether the 106 possible clusters available provided adequate power to test this hypothesis was conducted under the assumptions that on average the clusters will contribute 300 to 500 deliveries per year with a base mean rate of perinatal death of 40 to 50 per 1,000 deliveries and an intra-class correlation coefficient between 0.005 and 0.01 (based on preliminary data collected from these clusters). Using a two-sided hypothesis test at the 5% level of significance, these 106 clusters will provide a power of at least 80% across the full range of assumptions and greater than 90% across much of the range to detect a reduction of 30% in the perinatal mortality.

### Enrollment - Inclusion Criteria

The clusters have at least 300 deliveries per year with a minimum of 50% occurring at home or first level facilities. This is an intent-to-treat design. Thus all pregnancy outcomes of women who deliver in the study clusters and provide consent are collected.

### Enrollment - Exclusion Criteria

The intervention is intended to work by improving knowledge and affecting behavior change throughout pregnancy. Improved outcomes at delivery will depend on accrued benefits. Women who first move into the study area within 4 weeks of their actual delivery date are unlikely to derive adequate benefit from the intervention and will be excluded from the primary outcome.

### Randomization procedures

Preliminary data collected by the GN from these 106 clusters formed the basis for randomization. The DCC stratified clusters based on a cross-classification of birth and perinatal mortality rates and randomized within one to three comparable strata to intervention or control cluster within each Global Network site.

### Enrolment - Enlisting participants in the trial

The MNH Registry is an ongoing population-based pregnancy outcome data collection program in each of the clusters in the GN's 7 trial sites. The MNH Registry tracks all pregnancies, deliveries and delivery outcomes with its own staff which functions independently from the EMONC trial staff in order to reduce bias due to joint outcome data collection. The Registry in each cluster is managed by a Registry Administrator who, with assistants as needed, screens, enrolls, and tracks all pregnant women within the clusters. They obtain consent for data collection during pregnancy and collect delivery outcomes from all known pregnancies and all deaths of pregnant women up to and including 42 days post delivery/pregnancy termination, and 28-day neonatal outcomes. The Registry collects data during three visits to each mother including an enrollment visit as early as possible within pregnancy (targeted for 20 weeks gestation or greater), a second visit within 48 hours of delivery and a visit at day 42 after birth.

### Data Collection and Management

Outcome and process data are collected separately for the trial. Outcome data are collected through the MNH Registry utilizing standardized instruments to measure maternal demographic data, health care utilization, MNH outcomes and serious adverse events. Data collected for the EmONC trial implementation are collected using standardized instruments in the intervention clusters; process data include cluster team activities, community mobilization activities and training logs. Facility and community-based death audit forms support the activities of the facility trainers and community mobilization teams that utilize them for teaching and discussion.

All data are collected prospectively in the local language using hard copy forms, with only the individual study identification number on each form in order to maintain confidentiality. The forms are keyed into computers at in each country using a common data management system. At intervals ranging from daily to once per week, data are transmitted to the DCC for inclusion in the central database. The hard copy forms are retained in-country in a secure location.

## Discussion

Perinatal mortality and morbidity remains one of the largest challenges for improving maternal and child health in low and middle-income countries. Despite a good understanding of health interventions that can reduce this burden, sustained implementation has been a challenge. A comprehensive package of interventions spanning the continuum of care from the home to the hospital, with a robust community mobilization component, has the potential to significantly improve both maternal and fetal/newborn outcomes. The findings from the current RCT therefore may inform health policy for interventions that are scalable and sustainable in a variety of settings in LMIC.

## Competing interests

This project is being funded by the *Eunice Kennedy Shriver *National Institute of Child health and Human Development (NICHD), of the National Institutes of Health in the United States. The authors of this paper have no competing interests to declare.

## Authors' contributions

OP, LLW, RLG, SG, FA, and EMM conceived the study and participated in its design. DW participated in study design and the statistical analysis. SS, AP, FE, AG, MM, EC, RJD, PH, EAL, KMH, WAC, PB participated in the design of the study and oversight of the interventions. SS and LHG participated in the design and implementation of the community mobilization and birth attendant training components. All authors have reviewed and approved the final manuscript.

## Pre-publication history

The pre-publication history for this paper can be accessed here:

http://www.biomedcentral.com/1471-2393/10/82/prepub

## References

[B1] Ronsmans C Graham WJ Lancet Maternal Survival Series steering group. Maternal mortality: who, when, where, and why Lancet 2006 368 1189 200 10.1016/S0140-6736(06)69380-X 17011946

[B2] Stanton C Lawn JE Rahman H Wilczynska-Ketende K Hill K Stillbirth rates: delivering estimates in 190 countries Lancet 2006 367 1487 94 10.1016/S0140-6736(06)68586-3 16679161

[B3] Lawn JE Cousens S Zupan J Lancet Neonatal Survival Steering Team. 4 million neonatal deaths: When? Where? Why? Lancet 2005 365 891 900 10.1016/S0140-6736(05)71048-5 15752534

[B4] Hyder AA Wali SA McGuckin J The burden of disease from neonatal mortality: a review of South Asia and Sub-Saharan Africa BJOG 2003 110 894 901 10.1111/j.1471-0528.2003.02446.x 14550358

[B5] McClure EM Goldenberg RL Bann CM Maternal mortality, stillbirth and measures of obstetric care in developing and developed countries Int J Gynaecol Obstet 2007 96 139 46 10.1016/j.ijgo.2006.10.010 17274999

[B6] World Health Organization Perinatal mortality: a listing of available information FRH/MSM.96.7 1996 Geneva: WHO

[B7] National Health Survey of Pakistan Health profile of the people of Pakistan Islamabad, Pakistan: Pakistan Medical Research Council 1998

[B8] Sibley LM Sipe TA Brown CM Diallo MM McKatt K Habarta N Traditional birth attendant training for improving health behaviors and pregnancy outcomes (review) Cochrane Database of Systematic Review 2007 CD005460 10.1002/14651858.CD005460.pub2 17636799

[B9] Lawn J Shibuya K Stein C No cry at birth: global estimates of intrapartum stillbirths and intrapartum-related neonatal deaths Bull World Health Organ 2005 83 409 17 15976891 PMC2626256

[B10] Ronsmans C Severe acute maternal morbidity in low income countries Best Pract Res Clin Obstet Gynaecol 2009 23 305 16 10.1016/j.bpobgyn.2009.01.001 19201657

[B11] AbouZahr C Global burden of maternal death and disability Br Med Bull 2003 67 1 11 10.1093/bmb/ldg015 14711750

[B12] Lawn JE Kinney M Lee AC Chopra M Donnay F Paul VK Reducing intrapartum-related deaths and disability: Can the health system deliver? Int J Gynaecol Obstet 2009 107 Suppl 1 S123 40 S140-2. 10.1016/j.ijgo.2009.07.021 19815205

[B13] Campbell OM Graham WJ Lancet Maternal Survival Series steering group. Strategies for reducing maternal mortality: getting on with what works Lancet 2006 368 1284 99 10.1016/S0140-6736(06)69381-1 17027735

[B14] Penny S Murray SF Training initiatives for essential obstetric care in developing countries: a 'state of the art' review Health policy planning 2000 15 386 93 10.1093/heapol/15.4.386 11124241

[B15] Manandhar DS Osrin D Shrestha BP Mesko N Morrison J Tumbahangphe KM Members of the MIRA Makwanpur trial team. Effect of a participatory intervention with women's groups on birth outcomes in Nepal: cluster-randomised controlled trial Lancet 2004 364 970 9 10.1016/S0140-6736(04)17021-9 15364188

[B16] Jokhio AH Winter HR Cheng KK An intervention involving traditional birth attendants and perinatal and maternal mortality in Pakistan N Engl J Med 2005 352 2091 9 10.1056/NEJMsa042830 15901862

[B17] Thaddeus S Maine D Too far to walk: maternal mortality in context Soc Sci Med 1994 38 1091 110 10.1016/0277-9536(94)90226-7 8042057

[B18] Gabrysch S Campbell OM Still too far to walk: Literature review of the determinants of delivery service use BMC Pregnancy Childbirth 2009 9 34 10.1186/1471-2393-9-34 19671156 PMC2744662

[B19] Koblinsky M Matthews Z Hussein J Mavalankar D Mridha MK Anwar I Lancet Maternal Survival Series steering group. Going to scale with professional skilled care Lancet 2006 368 1377 86 10.1016/S0140-6736(06)69382-3 17046470

[B20] Filippi V Ronsmans C Campbell OM Graham WJ Mills A Borghi J Maternal health in poor countries: the broader context and a call for action Lancet 2006 368 2123 4 10.1016/S0140-6736(06)69384-7 17071287

[B21] UNICEF State of the World's Children 2009 2009 New York: UNICEF

[B22] Costello A Azad K Barnett S An alternative strategy to reduce maternal mortality Lancet 2006 368 2122 3 10.1016/S0140-6736(06)69388-4 17071268

[B23] Derman RJ Kodkany BS Goudar SS Geller SE Naik VA Bellad MB Oral misoprostol in preventing postpartum haemorrhage in resource-poor communities: a randomised controlled trial Lancet 2006 368 1248 53 10.1016/S0140-6736(06)69522-6 17027730

[B24] Paxton A Maine D Freedman L Fry D Lobis S The evidence for emergency obstetric care Int J Gynec Obstet 2005 88 181 93 10.1016/j.ijgo.2004.11.026 15694106

[B25] Chowdhury ME Ahmed A Kalim N Koblinsky M Causes of maternal mortality decline in Matlab, Bangladesh J Health Popul Nutr 2009 27 108 23 10.3329/jhpn.v27i2.3325 19489410 PMC2761779

[B26] Lubbock LA Stephenson RB Utilization of maternal health care services in the department of Matagalpa, Nicaragua Rev Panam Salud Publica 2008 24 75 84 10.1590/S1020-49892008000800001 19062598

[B27] Kerber KJ de Graft-Johnson JE Bhutta ZA Okong P Starrs A Lawn JE Continuum of care for maternal, newborn, and child health: from slogan to service delivery Lancet 2007 370 1358 69 10.1016/S0140-6736(07)61578-5 17933651

[B28] Primary health care Report of the International Conference on Primary Health Care, Alma-Ata, USSR, 6-12 September 1978, jointly sponsored by the World Health Organization and the United Nations Children's Fund 1978 Geneva: World Health Organization (Health for All Series, No. 1)

[B29] Arole M Arole R Taylor-Ide D, Taylor CE Jamkhed, India--the evolution of a world training center Just and lasting change: when communities own their futures 2002 Baltimore: Johns Hopkins University Press 150 60

[B30] Cueto M The ORIGINS of primary health care and SELECTIVE primary health care Am J Public Health 2004 94 1864 74 10.2105/AJPH.94.11.1864 15514221 PMC1448553

[B31] Filippi V Ronsmans C Campbell OM Graham WJ Mills A Borghi J Koblinsky M Osrin D Maternal health in poor countries: the broader context and a call for action Lancet 2006 368 1535 41 10.1016/S0140-6736(06)69384-7 17071287

[B32] Logie DE Rowson M Ndagije F Innovations in Rwanda's health system: looking to the future Lancet 2008 372 256 61 10.1016/S0140-6736(08)60962-9 18619670

[B33] Ensor T Clapham S Prasad PD What drives health policy formulation: Insights from the Nepal Maternity Incentive Scheme? Health Policy 2008 90 247 53 10.1016/j.healthpol.2008.06.009 19041153

[B34] Tsai TC Public health and peace building in Nepal Lancet 2009 374 515 6 10.1016/S0140-6736(09)61473-2 19691155

[B35] O'Rourke K Howard-Grabman L Seoane G Impact of community organization of women on perinatal outcomes in rural Bolivia Rev Panam Salud Publica 1998 3 9 14 10.1590/S1020-49891998000100002 9503957

[B36] Bang AT Reddy HM Deshmukh MD Baitule SB Bang RA Neonatal and infant mortality in the ten years (1993 to 2003) of the Gadchiroli field trial: effect of home-based neonatal care J Perinatol 2005 25 S92 107 10.1038/sj.jp.7211277 15791283

[B37] Tripathy P Nair N Barnett S Mahapatra R Borghi J Rath S Effect of a participatory intervention with women's groups on birth outcomes and maternal depression in Jharkhand and Orissa, India: a cluster-randomised controlled trial Lancet 2010 375 1182 92 10.1016/S0140-6736(09)62042-0 20207411

[B38] Azad K Barnett S Banerjee B Shaha S Khan K Rego AR Effect of scaling up women's groups on birth outcomes in three rural districts in Bangladesh: a cluster-randomised controlled trial Lancet 2010 375 1193 202 10.1016/S0140-6736(10)60142-0 20207412

[B39] Manandhar DS Osrin D Shrestha B Effect of a participatory intervention with women's groups on birth outcomes in Nepal: cluster-randomised controlled trial Lancet 2004 364 970 79 10.1016/S0140-6736(04)17021-9 15364188

[B40] Howard-Grabman L Snetro G How to mobilise communities for health and social change 2003 Baltimore, MD: Health Communication Partnership/USAID

[B41] Borghi J Thapa B Osrin D Jan S Morrison J Tamang S Economic assessment of a women's group intervention to improve birth outcomes in rural Nepal Lancet 2005 1882 4 10.1016/S0140-6736(05)67758-6 16310555

[B42] Anonymous MotherCare/Bolivia. MotherCare Matters 1999 8 8 16 http://www.mothercare.jsi.com/pubs/mcmatters/pdf/Vol8-2.pdf [MotherCare website on-line.] Cited May 31, 2006

[B43] Marsh DR Schroeder DG Dearden KA Sternin J Sternin M The power of positive deviance BMJ 2004 329 1177 9 10.1136/bmj.329.7475.1177 15539680 PMC527707

[B44] Berggren WL Wray JD Positive deviant behavior and nutrition education Food Nutr Bull 2002 23 9 10 12503226

[B45] Fullerton JT Killian R Gass PM Outcomes of a community and home-based intervention for safe motherhood and newborn care Health Care Women Int 26 561 576 10.1080/07399330591004881 16126600

[B46] Ensor T Cooper S Overcoming barriers to health service access: influencing the demand side Health Policy Plan 2004 19 69 79 10.1093/heapol/czh009 14982885

[B47] Stanton CK Methodological issues in the measurement of birth preparedness in support of safe motherhood Eval Rev 2004 28 179 200 10.1177/0193841X03262577 15130180

[B48] McPherson RA Khadka N Moore JM Sharma M Are birth-preparedness programmes effective? Results from a field trial in Siraha district, Nepal J Health Popul Nutr 2006 24 479 88 17591345 PMC3001152

[B49] Sibley LM Sipe TA Transition to skilled birth attendance: Is there a future role for trained traditional birth attendants? J Health Popul Nutr 2006 24 472 8 17591344 PMC3001151

[B50] Sibley L Sipe TA Koblinsky M Does traditional birth attendant training improve referral of women with obstetric complications: a review of the evidence Soc Sci Med 2004 59 1757 68 10.1016/j.socscimed.2004.02.009 15279931

[B51] Sibley LM Sipe TA Brown CM Diallo MM McNatt K Habarta N Traditional birth attendant training for improving health behaviours and pregnancy outcomes Cochrane Database of Systematic Reviews 2007 3 CD005460 10.1002/14651858.CD005460.pub2 17636799

[B52] Hofmeyr GJ Haws RA Bergström S Lee AC Okong P Darmstadt GL Mullany LC Oo EK Lawn JE Obstetric care in low-resource settings: what, who, and how to overcome challenges to scale up? Int J Gynaecol Obstet 2009 107 Suppl 1 S21 44 S44-5 10.1016/j.ijgo.2009.07.017 19815204

[B53] Darmstadt GL Bhutta ZA Cousens S Adam T Walker N de Bernis L Lancet Neonatal Survival Steering Team. Evidence-based, cost-effective interventions: how many newborn babies can we save? Lancet 2005 365 977 988 10.1016/S0140-6736(05)71088-6 15767001

[B54] Draycott T Sibanda T Owen L Akande V Winter C Reading S Whitelaw A Does training in obstetric emergencies improve neonatal outcome? BJOG 2006 113 177 82 10.1111/j.1471-0528.2006.00800.x 16411995

[B55] Sloan NL Nguyen TN Do TH Quimby C Winikoff B Fassihian G Effectiveness of lifesaving skills training and improving institutional emergency obstetric care readiness in Lam Dong, Vietnam J Midwif Womens Health 2005 50 315 23 10.1016/j.jmwh.2004.08.018 15973269

[B56] Pattinson R Kerber K Waiswa P Day LT Mussell F Asiruddin SK Blencowe H Lawn JE Perinatal mortality audit: counting, accountability, and overcoming challenges in scaling up in low- and middle-income countries Int J Gyn Obstet 2009 107 S113 21 10.1016/j.ijgo.2009.07.011 19815206

[B57] Carlo WA Goudar SS Jehan I Chomba E Tshefu A Garces A Parida S FIRST BREATH Trial. Newborn-care training and perinatal mortality in developing countries N Engl J Med 2010 362 614 23 10.1056/NEJMsa0806033 20164485 PMC3565382

[B58] Buffington S Sibley L Beck D Armbruster D Home Based Life Saving Skills 2004 1 Washington (DC): American College of Nurse Midwives

